# Metaverse in Mental Health: The Beginning of a Long History

**DOI:** 10.1007/s11920-024-01501-8

**Published:** 2024-04-11

**Authors:** Antonio Cerasa, Andrea Gaggioli, Giovanni Pioggia, Giuseppe Riva

**Affiliations:** 1 Institute for Biomedical Research and Innovation, National Research Council, IRIB-CNR, 98164 Messina, Italy; 2 S. Anna Institute, 88900 Crotone, Italy; 3 Pharmacotechnology Documentation and Transfer Unit, Preclinical and Translational Pharmacology, Department of Pharmacy, Health Science and Nutrition, University of Calabria, 87036 Arcavacata, Italy; 4 Research Center in Communication Psychology, Catholic University of Milan, Milan, Italy; 5 Applied Technology for Neuro-Psychology Lab, IRCCS Istituto Auxologico Italiano, Milan, Italy; 6 Humane Technology Lab, Catholic University of Milan, Largo Gemelli 1, 20123 Milan, Italy

**Keywords:** Metaverse, Predictive coding, Social brain, Brain-to-brain synchrony, Eating disorders, Sexual disorders, Autism spectrum disorders

## Abstract

**Purpose of Review:**

We review the first pilot studies applying metaverse-related technologies in psychiatric patients and discuss the rationale for using this complex federation of technologies to treat mental diseases. Concerning previous virtual-reality applications in medical care, metaverse technologies provide the unique opportunity to define, control, and shape virtual scenarios shared by multi-users to exploit the “synchronized brains” potential exacerbated by social interactions.

**Recent Findings:**

The application of an avatar-based sexual therapy program conducted on a metaverse platform has been demonstrated to be more effective concerning traditional sexual coaching for treating female orgasm disorders. Again, a metaverse-based social skills training program has been tested on children with autism spectrum disorders, demonstrating a significant impact on social interaction abilities.

**Summary:**

Metaverse-related technologies could enable us to develop new reliable approaches for treating diseases where behavioral symptoms can be addressed using socio-attentive tasks and social-interaction strategies.

## Introduction

The Metaverse, a term coined from the fusion of “meta” (meaning beyond) and “universe,” is usually defined as a hypothetical next-generation Internet that aims to create a shared virtual space connecting the physical and digital world [1, 2•]. This expansive, immersive digital environment offers users an opportunity to interact with a computer-generated universe, as well as with each other, in ways that mimic and extend real-world interactions [3].

The backbone of the Metaverse is built on some key technological tools [4], such as:

- Mixed reality (MR): This is an emerging technology that combines aspects of the real and virtual worlds to provide a range of experiences from fully virtual to wholly physical. MR is a computational environment that creates new habitats and representations where real-time interactions between digital and physical items take place. Both augmented reality (AR), which superimposes digital content in the real world, and virtual reality (VR), which submerges users in fully virtualized settings, are included.
- Artificial Intelligence (AI) algorithms play a crucial role in creating dynamic, interactive environments that can adapt and respond to user behavior, providing continuous probabilistic evaluation of user performance.
- Blockchain technology ensures secure, transparent transactions and ownership of digital assets, enabling a digital economy within the Metaverse.

**Fig. 1:**
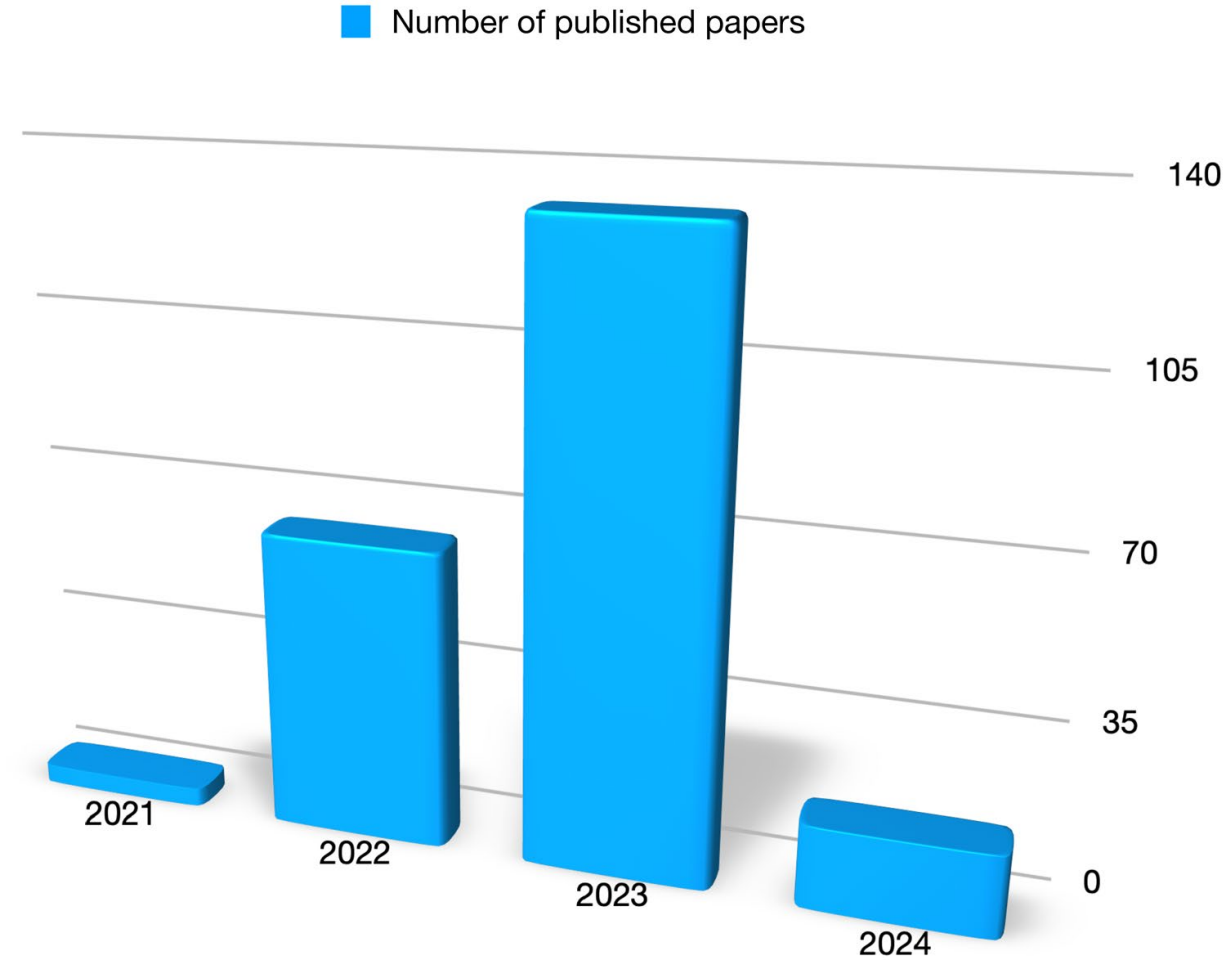
Number of published papers on the metaverse as reported on the PubMed database (access 06/02/2024), combining the terms “Metaverse” and “Health”

The scientific community has recently paid a great deal of attention to the Metaverse, and a great deal of work has already been done on its concepts, applications, and design. An exponential rise in the number of publications about metaverse can be seen while searching for them on PubMed between 2021 and the present (Fig. 1). Combining the terms “Health” and “Metaverse,” 206 publications have been published since the first original paper in March 2021, and the number is still rising.

However, despite this great scientific interest in the metaverse application in the health domain, only a few clinical trials have been opened in the last year (Table 1) (according to the clinicaltrials.gov website, all accessed on 06/02/2024).

**Table 1:** Clinical trials approved (clinicaltrials.gov website) on the application of the metaverse

**Title**	**NCT N°**
*The use of metaverse in nursing education*	NCT05829395
*Metaverse-based healthy life program for youth*	NCT05332886
*The effect of metaverse-based nursing skills laboratory*	NCT05706584
S*upport groups in the metaverse for Ukrainian refugees*	NCT06142032
*Positive youth development in the metaverse—a pilot study*	NCT05858593
*Effectiveness of metaverse space-based exercise video distribution in young adults*	NCT06019156
E*xamining the effect of metaverse-based epilepsy education*	NCT06195020
*Multidimensional rehabilitation intervention in colorectal cancer survivors*	NCT05956990
*Prevalence of internet addiction and its effects on psychological well-being of adults in Hong Kong*	NCT06205004
*Avatar-based therapy for female orgasmic disorder*	NCT06187246
*VR and script training of PWA*	NCT05667480

## Virtual Healing: Exploring the Metaverse’s Potential in Mental Health Care

As we have seen in the Introduction, the emerging Metaverse is envisioned as a blended (digital/physical) landscape that provides enhanced spaces for rich user interactions. Central to the Metaverse concept is a dynamic, two-way connection between the virtual and physical worlds. An example of this vision is the *digital twins*: digital representations of real-world entities—an object, system, or process—that are synchronized with the real world. This means that actions in the physical world can shape experiences in the virtual realm, and vice versa. Additionally, any alterations in the physical world are reflected in its digital twin, allowing for reciprocal feedback—for instance, interacting with an avatar in the virtual world can trigger haptic feedback in the real world.

This interconnectedness will be facilitated by the convergence and interaction of various digital technologies, including 3D shared XR (Extended Reality) environments, biosensors and activity sensors bridging real-world actions to virtual experiences, two-way Internet-of-Things (IoT) connections, social media platforms, and wearable technologies like smartphones. These tools work together to blur the lines between the digital and physical, enriching user experiences across both domains. The outcome of this process is “medical extended reality” (MXR), the use of Metaverse technologies to extend or enhance the medical experience [5, 6]. In this view, the application of the metaverse in mental health introduces a new paradigm in treatment methodologies, offering advantages that traditional approaches may lack.

First, the metaverse is distinguished by its ability to create controlled, safe, and customizable therapeutic environments, which can be tailored to the specific needs and conditions of individual patients [5]. The metaverse allows for the customization of therapeutic environments and scenarios to match the individual's specific needs, preferences, and treatment goals. This personalization is critical in mental health care, where the subjective experience of disorders necessitates tailored approaches for optimal outcomes.

Moreover, the connection between the virtual and physical worlds offers the capability for real-time monitoring and feedback [7], providing therapists with valuable data on patient progress and engagement levels. This immediate feedback, mediated by AI algorithms, could guide therapists in adjusting interventions more precisely to the patient’s needs, enhancing the effectiveness of treatment. Furthermore, immersive technologies, particularly through gamification and interactive scenarios, increase patient engagement and motivation [8]. This heightened level of involvement can lead to better adherence to treatment plans, as patients often find VR-based interventions more appealing and less stigmatizing than conventional therapies.

However, the Metaverse has another critical feature that provides a significant advantage to mental health: the metaverse shares with our brain the same basic mechanism—embodied simulations [9]. *Predictive coding* is an influential theory in neuroscience and cognitive science, proposing a framework for understanding how the brain processes information and interacts with the world [10]. Central to this approach is the idea that the brain is constantly generating and updating a model of the environment to predict sensory input, rather than passively receiving and reacting to external stimuli [11]. The predictive coding approach, with its emphasis on the brain’s predictive processes and error minimization, offers innovative perspectives and therapeutic strategies for improving mental health [12, 13]. By understanding mental disorders through the lens of disrupted or maladaptive predictive processes, this framework can guide the development of interventions aimed at recalibrating the brain’s prediction mechanisms, thus offering hope for more effective treatments.

In this view, the metaverse can be seen as a playground for predictive coding [14]. In fact, the metaverse works like our brain: it uses computer technology to predict and enact a simulated world that individuals can manipulate and explore as if they were in it [15]. Essentially, the metaverse seeks to replicate the sensory feedback one would anticipate from actual movements, presenting a simulated reality that adjusts dynamically to the user’s actions. The closer the metaverse aligns with our brain’s internal model, the more authentic and convincing the digital experience becomes, enhancing the user’s sense of presence within the virtual world. Moreover, the experiential potential of the metaverse can be used to design interventions aimed at recalibrating the brain’s predictive models to reduce prediction errors associated with mental health symptoms [1, 14]. By creating controlled environments, the metaverse can expose individuals to specific stimuli that challenge their maladaptive predictions, allowing for the safe correction of prediction errors.

## Applications in Psychiatric Domains: Exploiting the Brain-to-Brain Synchrony Potential

Exactly as it has already been demonstrated for VR [16], immersive technologies can aid clinicians in improving clinical assessment or exerting control over and modifying elements of the virtual environments that can be utilized to evaluate and test pertinent factors, including inducing social anxiety or monitoring reactions to environmental signals. However, concerning the large amount of evidence provided for the application of VR-related tools in mental health, the entry of the metaverse should be considered mainly for its social contents [9, 17]. As previously said, the metaverse represents a novel “federation” of several technologies featuring a robust service-oriented framework, prioritizing social content to establish connections between tangible and virtual realms [1]. The profound sense of presence that is shared in a social virtual setting is what makes a metaverse-related application effective. Because of this, individuals working on a virtual activity ought to do better when they collaborate compared to the traditional VR-related alone condition.

The social relevance of the metaverse-related applications relies on the theoretical background of the *social network theory* [18, 19]. This theory, which has its roots in psychology, sociology, and anthropology explains how social relationships, structures, and dynamics influence people’s attitudes, conduct, and overall well-being [20]. According to this theory, people are entangled in complex webs of relationships that include coworkers, friends, relatives, and other social groupings. During collective behaviors, people are engaged in exchanging knowledge about the object or purposes that the behavior is intended to achieve, accepting a common frame of reference and, finally, working together to accomplish it. These networks have a significant impact on a person’s mental health in addition to their behaviors and views.

However, connecting people in a social network to facing psychopathological thoughts or behaviors is the basis of the well-known group therapy approach, an old strategy used in clinical practice to strengthen interpersonal relationships in a group environment. Group therapy gives patients a safe space to explore feelings, obtain understanding, and create coping mechanisms through assisted interactions and shared experiences. In 2018, the American Psychological Association added group psychotherapy as a specialty, indicating that the scientific data supporting it is strong enough to qualify as an evidence-based treatment modality [21]. A plethora of studies demonstrated that group therapy is an effective method to treat a myriad of psychiatric disorders, such as mood [22], eating disorders [23], addiction [24], and schizophrenia [25].

Group therapy and social network theories have a complex interaction that provides important insights into the understanding and management of psychopathological diseases. First of all, for people who are struggling with mental health issues, social networks can be a source of stress as well as a resource. The quality, character, and structure of a person’s social network can have a big influence on how susceptible they are to psychopathological symptoms. For example, those with weak social support networks may be more susceptible to anxiety and depression, whereas people with strong social networks may benefit from improved mental health outcomes. Group therapy approaches aim to use the therapeutic potential of group dynamics within people’s social networks, thereby capitalizing on this understanding. Through the gathering of people with comparable experiences or obstacles, group therapy cultivates a feeling of acceptance and affirmation and gets help and inspiration in overcoming obstacles.

The neurophysiological basis underlying group therapy or social network approaches is the *brain-to-brain synchrony theory* [26, 27•]. This field of study explores the dynamic interplay between the brains of individuals engaged in social interactions. This theory suggests that during such interactions, there is a remarkable alignment of neural activity between individuals, leading to the synchronization of brain processes [28]. Through neuroimaging techniques (i.e., EEG), researchers have observed patterns of synchronized neural activity between individuals engaged in tasks requiring cooperation, communication, or empathy [29]. The phenomenon of brain-to-brain synchrony underscores the fundamental interconnectedness of human cognition and social behavior. It suggests that our brains are wired not only for individual processing but also for interpersonal coordination, allowing for shared understanding, empathy, and collaboration during collective tasks [30].

The brain-to-brain synchrony and the social network theories together with the group-therapy approach can be considered the neurocognitive background and the behavioral approach underlying the application of the metaverse in mental disorders. Exploiting the potential of the “synchronized brains” during socio-attentive functions increases the relevance of the social context and the planning of collaborative activities. This leads to a rise in interpersonal relationships, self-worth, and a deeper level of self-awareness [31, 32]. The metaverse could have the incredible potential to boost this effect by shaping and modulating multi-user virtual environments tailored to individual and group needs favoring the facilitator role of the therapist [2•].

## Metaverse Applications in Mental Disorders: The First Evidence

Here we present the first pilot studies applying metaverse-related technologies and approaches in mental disorders. In particular, three specific clinical fields have been explored: sexual dysfunctions, autism spectrum disorders, and eating disorders.

### Sexual Disorders

The first RCT study applying metaverse-related technology in mental disorders has been made by Vila et al. [33••]. They evaluated the efficacy of an avatar-based sexual therapy program for treating female orgasm disorder (FOD). This is a type of sexual dysfunction characterized by persistent or recurrent difficulty, delay, or absence of orgasm following sufficient sexual stimulation and arousal. This illness seriously affects women’s quality of life, interpersonal relationships, and sexual fulfillment. Estimates of FOD prevalence vary depending on the population studied and the criteria used for diagnosis; however, it has been reported that this disorder approximately affected 10–50% of older women [34, 35]. Several psychological and pharmacological therapeutic approaches have been proposed for addressing primary symptoms and improving sexual satisfaction, but their applications are often limited by psychosocial factors, such as guilt, shame, fear of exposure, and sexual stigma. For this reason, the employment of a metaverse-based approach could overcome these particular psychological barriers. As already stated by Riva et al. [36], using avatars has several therapeutic advantages, such as safety, flexibility, and access to virtual items and activities that could be difficult to reach in real life. In contrast to alternative remote or in-person therapies, metaverse-based therapy may also facilitate the use of therapeutic techniques like systematic desensitization, exposure therapy, and behavioral skills training (i.e., masturbation) by allowing patients’ avatars to confront anxiety-inducing stimuli and overcoming ethical concerns. For this reason, Vila et al. [33••] evaluated the feasibility and effectiveness of a new approach exploiting the metaverse-related approach applied to 31 women with FOD. The avatar-based psychotherapeutic program was conducted in the well-known metaverse Second Life platform (secondlife.com) and comprised twelve weekly online individual sessions where participants received a comprehensive 1-h daily treatment based on a combined cognitive-behavioral therapy (CBT) and the acceptance and commitment therapy (ACT) approaches. Otherwise, the control group sessions consisted of individual informative talks without the employment of specific therapeutic techniques. After the intervention, 100% of individuals enrolled in the experimental group fully recovered from FOD, including also increased sexual satisfaction, and lower sex guilt and anxiety. This result was explained as a consequence of avatar-based intervention which allows a more safe and easy access for examining genital parts of the body. Using a digital representation of yourself, it is possible to better alter one’s sense of one’s body while facilitating cognitive transformation. This kind of perceptual-cognitive task is difficult to perform during in-person or remote interventions due to ethical concerns. According to neuroscience, the body’s actions, thoughts, and emotions can be efficiently regulated and controlled by the brain through the generation of embodied simulations of the body in the real world, a process that the metaverse may have employed to create an embodied simulation of the body in the virtual world [37].

Despite this being the first study applying avatar-based intervention in sexual disorders, some aspects needed to be solved in order to fully exploit the metaverse-related potentials: (a) the employment of therapeutic sessions shared by multi-users using HMD and (b) the translation of cognitive and behavioral performance analyzed in a larger cohort to train a machine learning algorithm able to predict the clinical trajectory of patients during the daily sessions. These two particular advancements will allow us to exploit the social dimension of the metaverse-related experience promoting social learning resources and computational power through AI algorithms that can help to monitor and manage patients by automating data capture and sharing data across systems [38•].

### Neurodevelopmental Disorders

A collection of illnesses known as neurodevelopmental disorders (i.e., autism spectrum disorders and (ASD), attention-deficit/hyperactivity disorder (ADHD)) are defined by abnormalities in the growth and development of the central nervous system, which can lead to a variety of difficulties with cognition, emotion, and behavior. One common feature of many neurodevelopmental disorders is a deficiency in social functioning and social skills. Information and communication technologies (ICTs) have become more widely used in the past years to help people with ASD and ADHD regain social skills [39]. The rationale for applying a metaverse-related approach in neurodevelopmental disorders lies in the idea of exploiting the social dimensional experience of this technology on individuals with specific social skills deficits. The use of metaverse-related technologies may facilitate the application of behavioral therapies carried out gradually and individually under the direction of therapists, by exploiting creative environments where children may socialize using their imagination [40].

One of the first attempts to develop a metaverse-based social skills training program to enhance the capacity for social interaction has been launched by Lee et al. in 2022 [41] and terminated in 2023 [42••]. The metaverse game platform (Roblox) was employed to deliver the metaverse-based social skills training program (four 1-h sessions a week, for 4 total weeks) to 20 ASD individuals. With respect to traditional metaverse-related applications, in this program, the authors did not use HMD or avatar-related representations of their bodies. The four sessions made up the program. The introduction and knowledge of the necessity of guidelines and outcomes took place in the first session. Understanding the scenarios based on behavior and taking part in group activities were the focus of the second session. In the third session, the authors discussed how to react to unpleasant feelings and behavioral experiences. The topic of the fourth session was recognizing and valuing personal differences. Each session included homework, feedback, and metaverse practice in addition to theoretical sessions. Significant gains in social interaction abilities, improved mental health outcomes, a decrease in emotional and behavioral issues, and a reduction in parental psychological distress were all noted by the authors.

As concerns other neurodevelopmental disorders, such as ADHD, no pilot studies have been realized until now, although relevant guidelines to develop the first applications for improving social and learning disabilities in these individuals have been proposed [43].

### Eating Disorders

The APA’s DSM V designates eating disturbances as pivotal symptoms of eating disorders (EDs), emphasizing that these conditions hinge significantly on an individual’s relationship with their body [44–46]. This connection suggests that beyond the overt eating issues, a deeper concern with body image, encompassing the overemphasis on body shape and weight, is instrumental in understanding the roots of these disorders.

Although a metaverse-like tool has never been used in this specific clinical domain, several immersive technologies have been demonstrated to be effective in lowering symptoms and abnormal behaviors. We therefore present the most advanced VR/AR-related ED applications to encourage more translation within the metaverse’s social environment.

Recently, the Allocentric Lock Hypothesis (ALH) suggested that may EDs stem from deficits in multisensory body integration, a process that combines internal body signals with external information to create a coherent body experience [47–49]. Our body experience is not just visual. It is formed by integrating different sensory inputs like vision, touch, proprioception (body awareness), and interoception (internal signals like hunger, and fullness). This multisensory integration helps us create a coherent and updated representation of our physical selves. However, according to ALH, EDs may be caused by impairment in updating the internal multisensory representation of our body. In other words, ALH suggests that people with EDs become stuck in an observer-based (allocentric) view of their body, often shaped by negative judgments or self-objectifying experiences. Even if the individual’s body changes (e.g., losing weight after a diet) or they receive new sensory information (e.g., current visual and tactile input demonstrating that their body is underweight), the locked allocentric memory dominates. This inability to update leads to persistent body shame and dissatisfaction, even if it contradicts their current physical state. It fuels anxieties and unhealthy behaviors as attempts to control the perceived issue based on the locked memory, not the actual body. Furthermore, the inability to update their body image based on new experiences hinders them from accurately interpreting their internal signals related to emotions. To address this issue, as done with anxiety disorders, VR/AR systems are successfully used to extinguish/habituate craving [50] and anxiety responses to food‐related [51, 52] and body-related cues [53, 54].

Despite the development of exposure therapy strategies exploiting immersive technologies, a substantial volume of experimental research utilizing multisensory bodily illusions—such as the well-documented rubber hand illusion, where one feels a fake hand as part of their own body through synchronized stroking seen and felt on both the rubber and hidden real hand—to alter internal body representations by orchestrating multisensory discrepancies, particularly between sight and touch [55].

Technologies within the Metaverse can be leveraged to create full-body illusions, integrating the appearance of another individual’s body into one’s own body schema. This innovative technique involves the phenomenon of “inhabiting” a virtual body, achieved by synchronizing visual and tactile stimuli between the virtual and actual bodies from a first-person or a third-person viewpoint [56]. Recently, audio-only illusions were used, too [57], by altering in real-time the frequency components of the footsteps sounds produced by people as they walk, to make these sounds consistent with those produced by a lighter or heavier body. This innovative approach is being examined for its capacity to amend body misperceptions in individuals suffering from EDs. Through immersive technologies (e.g., virtual reality and auditory signals), these interventions aim to offer new therapeutic pathways by enabling users to experience and potentially reconcile distorted body images, offering promising directions for ED treatment and recovery [58].

Even if the research is still in its infancy, two different systematic reviews explored the potential of body illusions in modifying the bodily experience of clinical and non-clinical subjects [59, 60]. Both reviews underline that individuals with high Body Image Distortion (BID) show greater susceptibility to embodiment illusions. This suggests a potential link between BID and impairments in multisensory integration supporting the main claim of ALT. Second, the use of embodiment illusions can modify and enhance the flexible perception of individuals with Body Image Disturbance. This underscores the importance of integrating embodiment illusions into clinical environments as an adjunctive strategy alongside established treatments such as Cognitive Behavioral Therapy (CBT). Future research should combine illusions with technology targeting interoception to see if this improves effectiveness [61].

## Metaverse and Mental Health: Open Challenges

Before determining the true impact of metaverse-related applications for treating mental diseases in comparison to the well-known VR/AR technologies, we should wait a few years. Moreover, it should be borne in mind that the Metaverse will evolve into a prominent fixture in our digital lives; it brings with it a new dimension of mental health challenges. This immersive virtual world, while offering innovative opportunities for social interaction, entertainment, and education, also presents unique stressors that can impact users’ mental well-being. Understanding these challenges is crucial for developing effective strategies to promote mental health within these digital spaces.

The first challenge in the Metaverse relates to the concepts of reality and identity [62]. Users can create avatars that differ significantly from their real-world selves, enabling them to explore new identities and social dynamics. While this can offer a liberating form of self-expression and experimentation, it may also lead to issues related to identity confusion, especially among younger users who are still developing their sense of self. The blurred lines between one’s virtual and actual identity can complicate individuals’ self-perception and social interactions, potentially leading to dissociative experiences and psychological distress.

The Metaverse, as we have seen, has the potential to connect individuals across the globe, creating opportunities for interaction that transcend physical limitations [9]. However, this virtual connectivity might paradoxically lead to a second challenge: social isolation [63]. Users may prefer the company of virtual friends or communities, neglecting real-life relationships and interactions. This preference can exacerbate feelings of loneliness and isolation, as virtual interactions may lack the depth and emotional richness of face-to-face connections.

Moreover, the anonymity and distance provided by the Metaverse can embolden harmful behaviors such as cyberbullying and harassment [64]. Unlike the physical world, aggressors in the Metaverse can easily conceal their identities, making it difficult to hold them accountable for their actions. Victims of such behaviors may feel powerless and trapped, leading to significant emotional distress, anxiety, and depression.

Furthermore, the immersive nature of the Metaverse, with its engaging content and endless possibilities for exploration, can lead to addictive behaviors [65]. Users may spend excessive amounts of time in virtual environments, neglecting real-world responsibilities, relationships, and self-care. This escapism can serve as a coping mechanism for underlying issues such as depression, anxiety, or dissatisfaction with real life, further entrenching the individual in the virtual world and exacerbating mental health issues.

Finally, the Metaverse collects vast amounts of personal data to create personalized and immersive experiences. Concerns about privacy, data security, and surveillance can contribute to anxiety and paranoia. Moreover, the lack of control over personal information and the potential for its misuse can lead to feelings of vulnerability and distress, impacting users’ sense of psychological safety within these virtual spaces [1, 38•].

In conclusion, the Metaverse presents a complex landscape of mental health challenges that reflect the intricacies of human psychology in digital environments. By recognizing and addressing these issues, we can create a more inclusive, safe, and supportive Metaverse that enhances rather than detracts from users’ mental health.

## Conclusions

We can infer from these preliminary studies that the metaverse-related approach is currently working progress to mental health services. A new theoretical vision exploiting the potential of the brain during social interaction to overcome cognitive and emotional dysfunctions. However, to fully utilize the characteristics of this federation of technologies, numerous methodological advancements must be made while awaiting the preliminary results of the upcoming clinical trials. The question of which machine vision system is most appropriate for use in a healthcare context should be resolved first. Indeed, to satisfy the service’s demands about a deeper sense of immersion, we should move in the direction of the upcoming “phygital” technology paradigm, which refers to the possible blending of digital and physical places, by the convergence of virtual/augmented/mixed/extended reality, haptic device, Internet-of-Things, and AI [66]. For this reason, before beginning any new clinical studies, a great deal of work needed to be done to evaluate the usability of the upcoming devices.

Next, we should solve how, where, and whether AI-based algorithms can be employed in future metaverse-related tools. Indeed, with sensor-based wearable devices and other human–machine interaction tools, simple human movements and complex actions can be analyzed and recognized based on several machine learning or deep learning models [67]. However, before their application, we need a list of plausible biomarkers evaluating the efficacy of instruments associated with the metaverse. Stated differently, we must ascertain the precise correlation between digital biomarkers provided by MXR systems and the clinical metrics commonly utilized in clinical medicine [68]. Exactly as it has been made in the neurorehabilitation domain, where the exact relationship between robotic metrics and clinical scores has been described [69].

## Data Availability

No datasets were generated or analysed during the current study.

## References

[1] Cerasa A, Gaggioli A, Marino F, Riva G, Pioggia G (2022). The promise of the metaverse in mental health: the new era of MEDverse. Heliyon.

[2] Riva G, Wiederhold BK, Villani D (2024). Toward a humane metaverse: challenges and opportunities. Cyberpsychol Behav Soc Netw.

[3] Cheng R, Wu N, Varvello M, Chen S, Han B. Are we ready for metaverse? A measurement study of social virtual reality platforms. In Proceedings of the 22nd ACM Internet Measurement Conference (IMC ’22), October 25–27, Nice, France. ACM, New York, NY, USA, 2022:15 10.1145/3517745.3561417.

[4] Stylianos M (2022). Metaverse. Encyclopedia.

[5] Song YT, Qin J (2022). Metaverse and personal healthcare. Procedia Comput Sci.

[6] Plechatá A, Makransky G, Böhm R (2022). Can extended reality in the metaverse revolutionise health communication?. npj Digit Med.

[7] Ali S, Abdullah, Armand TPT, Athar A, Hussain A, Ali M, Yaseen M, Joo MI, Kim HC (2023). Metaverse in healthcare integrated with explainable ai and blockchain: enabling immersiveness, ensuring trust, and providing patient data security. Sensors.

[8] Flavián C, Ibáñez-Sánchez S, Orús C, Barta S (2024). The dark side of the metaverse: the role of gamification in event virtualization. Int J Inf Manage.

[9] Riva G, Wiederhold BK, Mantovani F (2024). Searching for the metaverse: neuroscience of physical and digital communities. Cyberpsychol Behav Soc Netw.

[10] Friston KJ (2018). Does predictive coding have a future?. Nat Neurosci.

[11] Apps MA, Tsakiris M (2014). The free-energy self: a predictive coding account of self-recognition. Neurosci Biobehav Rev.

[12] Smith R, Badcock P, Friston KJ (2021). Recent advances in the application of predictive coding and active inference models within clinical neuroscience. Psychiatry Clin Neurosci.

[13] Sterzer P, Adams RA, Fletcher P, Frith C, Lawrie SM, Muckli L, Petrovic P, Uhlhaas P, Voss M, Corlett PR (2018). The predictive coding account of psychosis. Biol Psychiat.

[14] Riva G, Serino S, Di Lernia D, Pagnini F (2021). Regenerative virtual therapy: the use of multisensory technologies and mindful attention for updating the altered representations of the bodily self. Front Syst Neurosci.

[15] Riva G, Di Lernia D, Sajno E, Sansoni M, Bartolotta S, Serino S, Gaggioli A, Wiederhold BK (2021). Virtual reality therapy in the metaverse: merging VR for the outside with VR for the inside. Annu Rev Cyberther Telemed.

[16] Bell IH, Nicholas J, Alvarez-Jimenez M, Thompson A, Valmaggia L (2020). Virtual reality as a clinical tool in mental health research and practice. Dialogues Clin Neurosci.

[17] Ford TJ, Buchanan DM, Azeez A, Benrimoh DA, Kaloiani I, Bandeira ID, Hunegnaw S, Lan L, Gholmieh M, Buch V, Williams NR (2023). Taking modern psychiatry into the metaverse: integrating augmented, virtual, and mixed reality technologies into psychiatric care. Front digit health.

[18] Wenlin L, Anupreet S, Amanda MB, Thomas V (2017). Social network theory. University of Southern California.

[19] Haslam SA, Jetten J, Postmes T, Haslam C (2009). Social identity, health and well-being: an emerging agenda for applied psychology. Appl Psychol.

[20] Valente W (2010). Social networks and health: models, methods, and applications.

[21] Whittingham M, Marmarosh CL, Mallow P, Scherer M (2023). Mental health care equity and access: a group therapy solution. Am Psychol.

[22] Janis RA, Burlingame GM, Svien H, Jensen J, Lundgreen R (2021). Group therapy for mood disorders: a meta-analysis. Psychother Res.

[23] Polnay A, James VA, Hodges L, Murray GD, Munro C, Lawrie SM (2014). Group therapy for people with bulimia nervosa: systematic review and meta-analysis. Psychol Med.

[24] Kaminer Y (2005). Challenges and opportunities of group therapy for adolescent substance abuse: a critical review. Addict Behav.

[25] Burlingame GM, Svien H, Hoppe L, Hunt I, Rosendahl J (2020). Group therapy for schizophrenia: a meta-analysis. Psychotherapy (Chic).

[26] Hamilton AF (2021). Hyperscanning: beyond the hype. Neuron.

[27] Shamay-Tsoory SG, Saporta N, Marton-Alper IZ, Gvirts HZ (2019). Herding brains: a core neural mechanism for social alignment. Trends Cogn Sci.

[28] Bhattacharya J (2017). Cognitive neuroscience: synchronizing brains in the classroom. Curr Biol.

[29] Hasson U, Ghazanfar AA, Galantucci B, Garrod S, Keysers C (2012). Brain-to-brain coupling: a mechanism for creating and sharing a social world. Trends Cogn Sci.

[30] Hari R, Kujala MV (2009). Brain basis of human social interaction: from concepts to brain imaging. Physiol Rev.

[31] Yamagishi A, Lee J, Sato N (2020). Oxytocin in the anterior cingulate cortex is involved in helping behaviour. Behav Brain Res.

[32] Ho SS, Macdonald A, Swain JE (2014). Associative and sensorimotor learning for parenting involves mirror neurons under the influence of oxytocin. Behav Brain Sci.

[33] Vila A, Ardoy-Cuadros J, Romero-Moreno R, Nogales-Gonzalez C, Ritchey AJ, Sansoni M, Riva G (2024). Body, emotions, and sexuality in the metaverse: a randomized control trial exploring the use of second life for an avatar-based intervention to support women with female orgasmic disorders. PsyArXiv Preprints.

[34] Shifren JL, Monz BU, Russo PA, Segreti A, Johannes CB (2008). Sexual problems and distress in United States women: prevalence and correlates. Obstet Gynecol.

[35] Brody S. Evaluation of female orgasmic disorder in W.W. IsHak (Ed.), The textbook of clinical sexual medicine (pp. 203–218). 2017. Springer International Publishing.

[36] Riva G, Malighetti C, Serino S (2021). Virtual reality in the treatment of eating disorders. Clin Psychol Psychother.

[37] Riva G, Wiederhold BK, Mantovani F (2019). Neuroscience of virtual reality: from virtual exposure to embodied medicine. Cyberpsychol Behav Soc Netw.

[38] Calabrò RS, Cerasa A, Ciancarelli I, Pignolo L, Tonin P, Iosa M, Morone G (2022). The arrival of the metaverse in neurorehabilitation: fact, fake or vision?. Biomedicines.

[39] Scarcella I, Marino F, Failla C, Doria G, Chilà P, Minutoli R, Vetrano N, Vagni D, Pignolo L, Di Cara M, Settimo C, Quartarone A, Cerasa A, Pioggia G (2023). Information and communication technologies-based interventions for children with autism spectrum conditions: a systematic review of randomized control trials from a positive technology perspective. Front Psychiatry.

[40] Spiel K, Frauenberger C, Keyes OS, Fitzpatrick G (2019). Agency of autistic children in technology research - a critical literature review. ACM Trans Comput Hum Interact.

[41] Lee J, Lee TS, Lee S, Jang J, Yoo S, Choi Y, Park YR (2022). Development and application of a metaverse-based social skills training program for children with autism spectrum disorder to improve social interaction: protocol for a randomized controlled trial. JMIR Res Protoc.

[42] Lee JH, Lee TS, Yoo SY, Lee SW, Jang JH, Choi YJ, Park YR (2023). Metaverse-based social skills training programme for children with autism spectrum disorder to improve social interaction ability: an open-label, single-centre, randomised controlled pilot trial. EClinicalMedicine.

[43] Mohamed A, Zohiar M, Ismail I. Metaverse and virtual environment to improve attention deficit hyperactivity disorder (ADHD) students’ learning. In: Frasson, C., Mylonas, P., Troussas, C. (eds) Augmented intelligence and intelligent tutoring systems. ITS 2023. Lec Notes Comput Sci. 2023;13891. Springer, Cham. 10.1007/978-3-031-32883-1_51.

[44] Brizzi G, Sansoni M, Riva G (2023). The BODY-FRIEND project: using new technology to learn about how people with anorexia feel about their bodies. Cyberpsychol Behav Soc Netw.

[45] Malighetti C, Sansoni M, Gaudio S, Matamala-Gomez M, Di Lernia D, Serino S, Riva G (2022). From virtual reality to regenerative virtual therapy: some insights from a systematic review exploring inner body perception in anorexia and bulimia nervosa. J Clin Med.

[46] Keizer A, Engel M. Body representation in anorexia nervosa. In The Routledge handbook of bodily awareness (pp. 380–397). 2022. Routledge.

[47] Riva G (2012). Neuroscience and eating disorders: the allocentric lock hypothesis. Med Hypotheses.

[48] Riva G, Gaudio S, Dakanalis A (2015). The neuropsychology of self objectification. Eur Psychol.

[49] Riva G, Dakanalis A (2018). Altered processing and integration of multisensory bodily representations and signals in eating disorders: a possible path toward the understanding of their underlying causes. Front Hum Neurosci.

[50] Nameth K, Brown T, Bullock K, Adler S, Riva G, Safer D, Runfola C (2021). Translating virtual reality cue exposure therapy for binge eating into a real-world setting: an uncontrolled pilot study. J Clin Med.

[51] Malighetti C, Schnitzer CK, YorkWilliams SL, Bernardelli L, Runfola CD, Riva G, Safer DL (2023). A pilot multisensory approach for emotional eating: pivoting from virtual reality to a 2-D telemedicine intervention during the COVID-19 pandemic. J Clin Med.

[52] Harris NM, Lindeman RW, Bah CSF, Gerhard D, Hoermann S (2023). Eliciting real cravings with virtual food: using immersive technologies to explore the effects of food stimuli in virtual reality. Front Psychol.

[53] Miquel-Nabau H, Briseño-Oloriz N, Porras-Garcia B, Ascione M, Meschberger-Annweiler F-A, Ferrer-Garcia M, Moreno-Sanchez M, Serrano-Troncoso E, Carulla-Roig M, Gutiérrez MJ (2023). Modification of body-related attentional bias through virtual reality and eye-tracking in healthy participants: implications for anorexia nervosa treatments. Brain Sci.

[54] Behrens SC, Tesch J, Sun PJ, Starke S, Black MJ, Schneider H, Giel KE (2023). Virtual reality exposure to a healthy weight body is a promising adjunct treatment for anorexia nervosa. Psychother Psychosom.

[55] Keizer A, Smeets MA, Postma A, van Elburg A, Dijkerman HC (2014). Does the experience of ownership over a rubber hand change body size perception in anorexia nervosa patients?. Neuropsychologia.

[56] Brizzi G, Sansoni M, Di Lernia D, Frisone F, Tuena C, Riva G (2023). The multisensory mind: a systematic review of multisensory integration processing in anorexia and bulimia nervosa. J Eat Disord.

[57] Tajadura-Jiménez A, Crucianelli L, Zheng R, Cheng C, Ley-Flores J, Borda-Más M, Bianchi-Berthouze N, Fotopoulou A (2022). Body weight distortions in an auditory-driven body illusion in subclinical and clinical eating disorders. Sci Rep.

[58] Portingale J, Krug I, Liu H, Kiropoulos L, Butler D. Your body, my experience: a systematic review of embodiment illusions as a function of and method to improve body image disturbance. medRxiv 2023;2023–10. 10.1101/2023.10.21.23297282.

[59] Portingale J, Krug I, Butler D (2024). Embodiment illusions and eating disorders: snapshot of implications for research and interventions. Trends Mol Med.

[60] Turbyne C, Goedhart A, de Koning P, Schirmbeck F, Denys D (2021). Systematic review and meta-analysis of virtual reality in mental healthcare: effects of full body illusions on body image disturbance. Front Virtual Real.

[61] cite Schoeller F, Horowitz AH, Jain A, Maes P, Reggente N, Christov-Moore L, Pezzulo G, Barca L, Allen M, Salomon R, Miller M, Di Lernia D, Riva G, Tsakiris M, Chalah MA, Klein A, Zhang B, Garcia T, Pollack U, Trousselard M, Verdonk C, Dumas G, Adrien V, Friston K. Interoceptive technologies for psychiatric interventions: From diagnosis to clinical applications. Neurosci Biobehav Rev. 2024 Jan;156:105478. 10.1016/j.neubiorev.2023.105478. 10.1016/j.neubiorev.2023.105478 38007168

[62] Riva G, Wiederhold BK (2022). What the metaverse is (really) and why we need to know about it. Cyberpsychol Behav Soc Netw.

[63] Novak R (2022). The rise of the ‘immersive virtual online avatar society’: does an online community established in the virtual space constitute a ‘real’society?: visual technologies as a panacea for social isolation. Video J Educ Pedagogy.

[64] Wiederhold BK (2022). Sexual harassment in the metaverse. Cyberpsychol Behav Soc Netw.

[65] Barreda-Ángeles M, Hartmann T (2022). Hooked on the metaverse? Exploring the prevalence of addiction to virtual reality applications. Frontiers in virtual reality.

[66] Gaggioli A, Cerasa A, Barresi G. Phygital mental health: opportunities and challenges. In: Scataglini, S., Imbesi, S., Marques, G. (eds) mHealth and human-centered design towards enhanced health, care, and well-being. Stud Big Data. 2023;120. Springer, Singapore. 10.1007/978-981-99-3989-3_2.

[67] Huynh-The T, Pham QV, Pham XQ, Nguyen TT, Han Z, Kim DS. Artificial intelligence for the metaverse: a survey. Eng Appl Artif Intell 2023;117(5):105581. 10.1016/j.engappai.2022.105581.

[68] Riva G, Wiederhold BK, Di Lernia D, Chirico A, Riva EFM, Mantovani F, Cipresso P, Gaggioli A (2019). Virtual reality meets artificial intelligence: the emergence of advanced digital therapeutics and digital biomarkers. Annu Rev Cyberther Telemed.

[69] Tran VD, Dario P, Mazzoleni S (2018). Kinematic measures for upper limb robot-assisted therapy following stroke and correlations with clinical outcome measures: a review. Med Eng Phys.

